# Evaluation of Elvitegravir/Cobicistat/Emtricitabine/Tenofovir Alafenamide in Treatment-Naive Patients Living With Human Immunodeficiency Virus (HIV) With High Viral Load: A Retrospective Observational Study

**DOI:** 10.7759/cureus.112191

**Published:** 2026-07-07

**Authors:** Hazel Öztürk Belik, Sümeyra Şimşek, Esra Kazak, Harun Ağca, Yasemin Heper, Ferah Budak, Emel Yılmaz, Haluk Barbaros Oral, Halis Akalın

**Affiliations:** 1 Infectious Diseases and Clinical Microbiology, Bursa Uludag University Faculty of Medicine, Bursa, TUR; 2 Infectious Diseases and Clinical Microbiology, Kanuni Sultan Süleyman Training and Research Hospital, Istanbul, TUR; 3 Medical Microbiology, Bursa Uludag University Faculty of Medicine, Bursa, TUR; 4 Immunology, Bursa Uludag University Faculty of Medicine, Bursa, TUR

**Keywords:** acquired immune deficiency syndrome (aids), antiretroviral therapies, elvitegravir, hiv integrase strand transfer inhibitors (insti), human immune deficiency virus, viral load

## Abstract

Introduction

Data on the long-term real-world efficacy of elvitegravir/cobicistat/emtricitabine/tenofovir alafenamide (E/C/F/TAF) in people living with HIV (PLWH) presenting with high baseline viral loads remain limited. This study aimed to assess the virological and immunological efficacy of E/C/F/TAF in treatment-naive patients stratified by baseline viremia levels.

Methods

This retrospective, single-center, observational study evaluated 67 treatment-naive adult patients who initiated E/C/F/TAF therapy between 2017 and 2018. Patients were stratified into three baseline viral load strata: low (<100,000 copies/mL; n=41), high (100,000 to <1,000,000 copies/mL; n=15), and very high (≥1,000,000 copies/mL; n=11). Virological suppression kinetics and absolute CD4+ T cell counts were longitudinally analyzed over a 24-month follow-up period using available-case analysis.

Results

Overall virological suppression was achieved in 85.1% (57/67) of patients at month six. Stratified analysis at month six revealed a statistically significant difference in early suppression rates: 97.6% (40/41) in the low, 73.3% (11/15) in the high, and 54.5% (6/11) in the very high strata (p<0.001). However, this disparity progressively narrowed; by month 12, suppression rates reached 100.0% (40/40) in the low, 86.7% (13/15) in the high, and 90.9% (10/11) in the very high strata (p=0.06), maintaining statistical similarity through month 24 (p=0.16), where 100.0% of the low (40/40) and high (14/14) strata, and 90.0% (9/10) of the very high stratum achieved suppression. Only one patient (baseline viral load: 3,393,215 copies/mL) exhibited persistent low-level viremia, which gradually declined to 114 copies/mL at month 12 and 54 copies/mL at month 24. Immunologically, the mean absolute CD4+ T cell count for the entire cohort expanded significantly from 414.1±254.8 cells/µL at baseline to 730.4±308.3 cells/µL at month 24. Linear mixed-effects modeling demonstrated a significant overall increase in CD4+ counts over time (p<0.001) and a significant time-by-group interaction (p=0.013), confirming an accelerated 'catch-up' immune recovery in the very high viral load stratum by month 24.

Conclusions

E/C/F/TAF was associated with durable long-term virological and immunological responses in treatment-naive PLWH, including those with high baseline viremia. Although high pre-treatment viral load was associated with a transient delay in early viral suppression at month six, this did not appear to compromise longer-term treatment outcomes. These findings suggest that E/C/F/TAF may represent a useful treatment option, particularly in clinical settings where second-generation integrase inhibitors are restricted or unavailable.

## Introduction

Human immunodeficiency virus-1 (HIV-1) treatment guidelines recommend initiating antiretroviral treatment (ART) with an integrase strand transfer inhibitor (INSTI), a protease inhibitor (PI), or a non-nucleoside reverse transcriptase inhibitor (NNRTI) in combination with a nucleoside reverse transcriptase inhibitor (NRTI) backbone as the preferred initial regimen [1,2]. Tenofovir alafenamide (TAF) is one of the recommended NRTIs. TAF in combination with elvitegravir/cobicistat/emtricitabine (E/C/F/TAF) has been shown to achieve a virologic success rate of 92% at 48 weeks in treatment-naive people living with HIV (PLWH) [3].

Phase III clinical trials demonstrated that E/C/F/TAF possessed non-inferior efficacy at weeks 48 and 96, and was superior to elvitegravir/cobicistat/emtricitabine/tenofovir disoproxil fumarate (E/C/F/TDF) at week 144. Furthermore, there was significantly less impact on renal biomarkers and bone mineral density [3-5]. Although its therapeutic profile is well-established, the proportion of patients presenting with high baseline viral loads varies considerably across clinical trials and real-world cohorts. Wohl et al. reported that only 5.2% of treatment-naive patients who received E/C/F/TAF had a baseline viral load >400,000 copies/mL in their study [5]. Conversely, Sax et al. reported that 23% (n=196) of the treatment-naive patients who received E/C/F/TAF had an HIV-1 ribonucleic Acid (RNA) concentration >100,000 copies/mL [3]. Korten et al. reported that 42.4% (n=253) of the treatment-naive patients had an HIV-1 RNA concentration >100,000 copies/mL in their real-world study from Türkiye [6].

While newer unboosted INSTI-based regimens, such as bictegravir- or dolutegravir-based combinations, are currently preferred as first-line therapies globally [1,2], older single-tablet regimens like E/C/F/TAF retain clinical relevance under specific circumstances. First, procurement delays, supply chain interruptions, or regional reimbursement barriers can limit the immediate availability of the newest preferred initial treatments. Second, patient-specific factors, including drug intolerances or specific contraindications to newer agents, may preclude their use. Furthermore, in settings where two-drug regimens (such as dolutegravir plus lamivudine) are readily available but concerns persist regarding their efficacy in patients presenting with high baseline viral loads, a robust three-drug regimen like E/C/F/TAF offers a viable therapeutic alternative to mitigate the risk of virological non-suppression.

Data on the use of E/C/F/TAF in treatment-naive PLWH with extreme baseline viremia (HIV-1 RNA ≥1,000,000 copies/mL) remain limited. To address this gap in a real-world setting, we evaluated a cohort treated at the Bursa Uludag University Faculty of Medicine Hospital. Specifically, this study aimed to evaluate the long-term virological and immunological efficacy of E/C/F/TAF in treatment-naive PLWH stratified by baseline viral load levels over a 24-month follow-up period.

## Materials and methods

Data collection and study design

This retrospective, single-center, observational study utilized consecutive sampling. The final sample size (n=67) encompassed all consecutive, treatment-naive adult PLWH aged ≥18 years who initiated an E/C/F/TAF regimen at our university hospital between 2017 and 2018. This non-probabilistic census approach ensured that the study population fully represented the institutional clinical registry without selection bias.

The study was conducted in accordance with the Declaration of Helsinki and approved by the Bursa Uludag University Faculty of Medicine Ethics Committee (Approval No: 2022-2/4). Clinical findings, baseline viral loads, and longitudinal CD4+ T cell counts were retrospectively extracted from medical records. Following ART initiation, routine clinical and laboratory follow-ups occurred at months two, six, and every six months thereafter, with months six, 12, and 24 predefined as major milestones for long-term endpoints. Medication adherence was routinely evaluated during clinical visits through patient interviews and verified via electronic pharmacy refill records.

Clinical staging was performed using the Centers for Disease Control and Prevention (CDC) 1993 revised classification system [7] and the World Health Organization (WHO) clinical staging criteria [8]. Plasma HIV-1 RNA levels were quantified using a real-time polymerase chain reaction assay - RealArt HIV-1 RG RT-PCR Kit (Artus GmbH, Germany) with a lower limit of quantification (LLoQ) of 70 IU/mL, corresponding to 40.6 copies/mL using the standard 0.58 conversion factor. Absolute CD4+ T cell counts were quantified via flow cytometry utilizing the FlowCount method (Beckman Coulter, United Kingdom).

Statistical analysis

The primary endpoint was to compare virological suppression rates-defined as an HIV-1 RNA level below the LLoQ (<40.6 copies/mL)-at months six, 12, and 24 among patients stratified into three baseline strata: low (<100,000 copies/mL), high (100,000 to <1,000,000 copies/mL), and very high (≥1,000,000 copies/mL). Missing data across follow-up milestones were managed using an available-case (on-treatment) analysis approach, restricting the denominator at each specific milestone to patients with available laboratory documentation.

Categorical variables are presented as frequencies and percentages (%). Continuous variables are summarized as mean±standard deviation (SD). Rates of viral suppression were calculated with corresponding 95% confidence intervals (CIs) using the Wilson score method. Longitudinal comparisons among the three strata were performed using Fisher’s exact test (Fisher-Freeman-Halton extension for 3x2 tables) due to expected cell counts less than five across milestones.

To evaluate the independent effect of baseline viral load on month six suppression, multivariable logistic regression analysis was conducted using the Enter method. Chronological age, baseline CD4+ T cell count, and a history of opportunistic infections were included as clinical covariates. Biological sex was excluded from the final matrices due to a perfect separation artifact (100% of female patients achieved suppression by month six) to safeguard model convergence. Baseline viremia was adjusted using two configurations: as a tri-categorical variable with the low viral load group as the reference (Model A), and as a continuous covariate via log10 transformation (Model B). Multicollinearity was assessed using the variance inflation factor (VIF).

Longitudinal trajectories of absolute CD4+ T cell counts at baseline, month six, month 12, and month 24 across the three baseline viral load strata were compared using a linear mixed-effects model (LMM) to account for within-subject correlations and missing data across follow-up milestones.

Analyses were performed using SPSS Statistics, Version 25.0 (IBM Corporation, Armonk, USA). A two-sided p-value <0.05 was considered statistically significant.

## Results

A total of 71 treatment-naive patients were enrolled in the study. Three patients were excluded due to missing data at month six, and one patient was excluded owing to non-adherence to ART. Ultimately, 67 patients were included in the final analysis.

The majority of the cohort consisted of male patients (n=60, 89.6%). The age of the participants ranged from 21 to 64 years, with a mean age of 36.4±9.9 years. Detailed demographic, epidemiological, and clinical characteristics of the cases are presented in Table 1.

**Table 1:** Demographic, epidemiological and clinical characteristics of patients Data are presented as frequencies with percentages [n (%)] for categorical variables, and as mean±SD or minimum–maximum ranges for continuous variables. SD: standard deviation; HIV: human immunodeficiency virus;  HIV-1 RNA: human immunodeficiency virus type 1 ribonucleic acid.

Characteristics of Patients	
Age, Mean±SD	36.4±9.9
Gender, n (%)	
Male	60 (89.6%)
Female	7 (10.4%)
Mode of Transmission, n (%)	
Sexual Contact	51 (76.1%)
-Men Who Have Sex with Men	12 (17.9%)
-Heterosexual	34 (50.7%)
-Unknown	5 (7.5%)
Intravenous Drug Use	2 (3%)
Blood Transfusion	1 (1.5%)
Other	5 (7.5%)
Unknown	8 (11.9%)
Reason for Testing, n (%)	
Clinical Suspicion	25 (37.3%)
Screening Before Blood Donation	7 (10.4%)
Pre-operative Screening	3 (4.5%)
Premarital Screening	3 (4.5%)
HIV-positive Spouse	5 (7.5%)
Patient’s Request	7 (10.4%)
Referral from an External Center for Treatment	12 (17.9%)
Other	5 (7.5%)
Comorbidities, n (%)	
Chronic Hepatitis C	1 (1.5%)
Chronic Hepatitis B	4 (5.9%)
Syphilis	6 (8.9%)
Hypertension	3 (4.5%)
Diabetes Mellitus	2 (3%)
Coronary Artery Disease	1 (1.5%)
Baseline HIV-1 RNA, copies/mL	
Mean±SD	602,187±1,600,843
Minimum–Maximum	460–11,580,330
HIV-1 RNA, n (%)	
<100,000 copies/mL	41 (61.2%)
100,000 to <1,000,000 copies/mL	15 (22.4%)
≥1,000,000 copies/mL	11 (16.4%)
CD4+ T Cell Count, cells/µL	
Mean±SD	414±255
Minimum–Maximum	8–1140
CD4+ T cell count, n (%)	
<200 cells/µL	16 (23.9%)
≥200 cells/µL	51 (76.1%)

At the time of diagnosis, acute retroviral syndrome was present in five patients (7.5%). According to the CDC 1993 revised classification system [7], the cohort was primarily distributed across Category A2 (CD4+ T cell count 200-499 cells/µL without AIDS-defining conditions: 43.3%, n=29), Category A1 (CD4+ T cell count >500 cells/µL without AIDS-defining conditions: 32.8%, n=22), and Category C3 (CD4+ T cell count <200 cells/µL with AIDS-defining conditions: 14.9%, n=10). Furthermore, based on the WHO clinical staging system [8], 77.6% (n=52) of the patients were classified as clinical stage 2, while 10.4% (n=7) presented in clinical stage 4.

Baseline HIV-1 RNA levels in the 67 treatment-naive patients initiated on E/C/F/TAF ranged from 460 to 11,580,330 copies/mL, with a mean±SD of 602,187±1,600,843 copies/mL. At the end of month six, HIV-1 RNA was undetectable in 85.1% (57/67) of the patients. The cumulative suppression rate increased to 95.5% (63/66) at month 12. By month 24, the final available-case suppression rate reached 98.4% (63/64).

**Figure 1:**
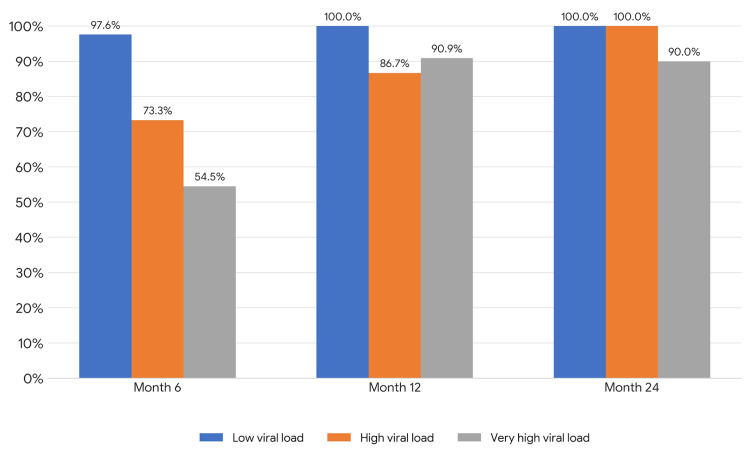
Virologic suppression rates by baseline HIV-1 RNA level Patients were categorized into three baseline viral load strata: Low (<100,000 copies/mL), High (100,000 to <1,000,000 copies/mL), and Very High (≥1,000,000 copies/mL). Virological suppression was defined as an HIV-1 RNA level <70 IU/mL (40.6 copies/mL). Percentages are calculated using an available-case (on-treatment) approach at each follow-up milestone (months six, 12, and 24). HIV-1 RNA: human immunodeficiency virus type 1 ribonucleic acid.

At ART initiation, patients were stratified into three distinct groups based on their baseline viral load, and their longitudinal virological suppression rates are illustrated in Table 2 and Figure 1. Among patients with a baseline HIV-1 RNA <100,000 copies/mL (n=41), virological suppression was 97.6% at month six, with complete suppression achieved by month 12. In the high baseline stratum (n=15), suppression progressed from 73.3% at month six to 86.7% at month 12, reaching 100.0% by month 24. For the very high baseline stratum (≥1,000,000 copies/mL, n=11), the suppression rate increased from 54.5% at month six to 90.9% at month 12. This rate was maintained at 90.0% among evaluable cases at month 24. The single patient with persistent detectable viral load at month 24 had a baseline HIV-1 RNA level of 3,393,215 copies/mL, which decreased to 114 copies/mL at month 12 and 54 copies/mL at month 24. Pre-treatment genotypic resistance analysis revealed no drug-resistance mutations, and no post-treatment resistance testing was performed. This patient demonstrated excellent treatment adherence and tolerated the regimen well. 

**Table 2:** Longitudinal viral suppression rates over time based on baseline viral load strata *Due to the presence of expected cell counts less than 5 across all evaluated time points, Fisher's exact test (Fisher-Freeman-Halton extension for 2x3 tables) was systematically utilized to determine statistically significant differences in virological suppression rates between the baseline viral load strata. Note: An On-Treatment approach was used. At month 12, data from 1 patient and at month 24, data from 3 patients (1 from each group) were missing; thus, percentages for month 24 are based on the remaining 64 evaluable patients. VL: viral load; CI: confidence interval (calculated using the Wilson score method).

Follow-up Period	Low Baseline VL (<100,000 copies/mL)	High Baseline VL (100,000 to <1,000,000 copies/mL)	Very High Baseline VL (≥1,000,000 copies/mL)	Total Cohort	p-value*
Month 6	97.6% (40/41) (95% CI: 87.4– 99.6)	73.3% (11/15) (95% CI: 48.0–89.1)	54.5% (6/11) (95% CI: 28.0–78.7)	85.1% (57/67) (95% CI: 74.7–91.7)	<0.001
Month 12	100.0% (40/40) (95% CI: 91.2–100.0)	86.7% (13/15) (95% CI: 62.1–96.3)	90.9% (10/11) (95% CI: 62.3–98.4)	95.5% (63/66) (95% CI: 87.5–98.4)	0.06
Month 24	100.0% (40/40) (95% CI: 91.2–100.0)	100.0% (14/14) (95% CI: 78.5–100.0)	90.0% (9/10) (95% CI: 59.6–98.2)	98.4% (63/64) (95% CI: 91.7–99.7)	0.16

When patients receiving E/C/F/TAF were stratified according to their baseline viral loads, a statistically significant difference was observed in early (month six) virological suppression rates (p<0.001). However, this disparity narrowed over time; at month 12 (p=0.06) and month 24 (p=0.16), the virological suppression rates between the groups were statistically similar.

To identify the independent predictors of virological suppression, multivariable logistic regression analysis was performed utilizing two distinct modeling approaches for baseline viral burden (Table 3). In Model A, the pre-treatment HIV-1 RNA level was analyzed as a categorical variable. Compared to the reference category of patients with a low baseline viral load (<100,000 copies/mL), those within the high baseline viral load stratum (100,000 to <1,000,000 copies/mL) demonstrated a borderline reduction in the odds of achieving virological suppression at month six, which did not reach statistical significance. This independent negative impact on early viral clearance kinetics was statistically significant and more pronounced among patients in the very high baseline viral load stratum (≥1,000,000 copies/mL), who exhibited a substantial reduction in the odds of virologic suppression. Conversely, chronological age, a history of opportunistic infections, and absolute baseline CD4+ T cell counts did not exert a statistically significant independent effect on early suppression. In Model B, the independent influence of baseline viremia was further validated by treating pre-treatment HIV-1 RNA as a continuous variable alongside immune markers. Every 1-log_10_ copies/mL increase in baseline viral load was found to be independently associated with significantly lower odds of achieving an undetectable viral load at month six. Consistent with the categorical model, chronological age, a history of opportunistic infections, and absolute baseline CD4+ T cell counts demonstrated no independent statistical association with early virological suppression.

**Table 3:** Multivariable logistic regression models predicting viral suppression at month 6 Note: Gender was excluded from both models due to complete quasi-separation (all female patients achieved virological suppression at month 6). B: unstandardized regression coefficient (or coefficient); S.E.: standard error; df: degrees of freedom; Sig.: statistical significance (p-value); OR: odds ratio; CI: confidence interval; VL: viral load; Ref: reference category; HIV-1 RNA: human immunodeficiency virus type 1 ribonucleic acid.

	B	S.E.	Wald	df	Sig. (p)	Exp(B) (OR)	95% CI for Exp(B) (Lower–Upper)
Model A: Categorical Baseline Viral Load							
Baseline VL Strata (Overall)			5.43	2	0.07		
High vs. Low (Ref)	-2.34	1.21	3.76	1	0.05	0.10	0.01–1.02
Very High vs. Low (Ref)	-2.89	1.26	5.27	1	0.02	0.06	0.00–0.65
Age	-0.01	0.04	0.05	1	0.82	0.99	0.92–1.07
Opportunistic Infection	-0.75	1.12	0.44	1	0.51	0.47	0.05–4.27
Baseline CD4+ T Cell Count	0.00	0.00	1.14	1	0.29	1.00	1.00–1.01
Constant (Intercept)	3.15	2.06	2.33	1	0.13	23.24	
Model B: Continuous Baseline Viral Load							
log_10 _Baseline HIV-1 RNA	-1.01	0.48	4.40	1	0.04	0.36	0.14–0.94
Age	-0.01	0.04	0.04	1	0.84	0.99	0.92–1.07
Opportunistic Infection	-0.98	1.11	0.77	1	0.38	0.38	0.04–3.35
Baseline CD4+ T Cell Count	0.00	0.00	1.80	1	0.18	1.00	1.00–1.01
Constant (Intercept)	6.60	3.22	4.21	1	0.04	737.15	

Regarding the immunological response, the mean absolute CD4+ T cell count for the entire cohort increased from 414.1±254.8 cells/µL at baseline to 730.4±308.3 cells/µL at month 24. In the subgroup with a very high baseline viral load (≥1,000,000 copies/mL), the mean CD4+ T cell count increased from 238.4±224.9 cells/µL at baseline to 725.6±414.4 cells/µL at month 24 (Table 4). Linear mixed-effects model analysis demonstrated a highly significant overall main effect of time for CD4+ recovery (p<0.001). A statistically significant Time x Group interaction effect was noted (p=0.012), indicating significantly different rates of CD4+ T cell recovery among the baseline strata, with the very high baseline viral load stratum exhibiting an accelerated recovery that resulted in absolute CD4+ T cell counts statistically comparable to the lower baseline strata by month 24.

**Table 4:** Longitudinal absolute CD4+ T cell counts based on baseline viral load groups (cells/µL) Data are expressed as mean±standard deviation. Note: Group sample sizes (n) indicate the number of patients at baseline. Due to missing data during follow-up, the available number of patients varied as follows: at month 12 (Low: n=40, High: n=15, Very High: n=10; total n=65) and at month 24 (Low: n=40, High: n=13, Very High: n=10; total n=63).

Viral Load Group	Baseline CD4+ T cell counts	Month 6 CD4+ T cell counts	Month 12 CD4+ T cell counts	Month 24 CD4+ T cell counts
Low (<100,000 copies/mL) (n=41)	474.8±220.6	632.8±270.4	676.7±236.7	727.9±294.6
High (100,000 to <1,000,000 copies/mL) (n=15)	376.9±307.1	538.5±256.0	594.7±295.2	741.6±282.7
Very High (≥1,000,000 copies/mL) (n=11)	238.4±224.9	467.5±198.1	534.3±218.7	725.6±414.4
Total Cohort	414.1±254.8	584.5±261.5	635.9±251.0	730.4±308.3

## Discussion

In treatment-naive PLWH, the initiation of combination ART containing INSTIs characteristically yields a rapid and profound decline in plasma viral load. Both the Department of Health and Human Services (DHHS) and the European AIDS Clinical Society (EACS) guidelines systematically recommend a regimen consisting of two NRTIs coupled with one potent INSTI as a preferred initial therapeutic strategy, irrespective of the patient's baseline viremia level [1,2]. The present study evaluated the real-world clinical efficacy of E/C/F/TAF in a cohort of 67 treatment-naive patients, with a specific emphasis on individuals presenting with high or very high baseline viral loads. Over the 24-month follow-up period, robust virological suppression and substantial immunological restoration were achieved across the cohort, demonstrating strong alignment with expanding national real-world data evaluating E/C/F/TAF dynamics [6,9,10].

Inan et al. investigated the real-world efficacy of E/C/F/TAF among treatment-naive patients presenting with extreme viremia, conducting a pairwise comparison between individuals with a very high baseline viral load (≥1,000,000 copies/mL; n=17) and the remainder of the cohort (n=150). Employing a suppression threshold of <200 copies/mL, they reported that early virological suppression rates at month six were significantly lower in the very high viral load group compared to patients with lower baseline viremia (76.5% vs. 96.0%, p<0.001) [9]. Santoro et al. [11] similarly stratified 1,430 treatment-naive patients into distinct baseline viral load strata (<30,000, 30,001-100,000, 100,001-300,000, 300,001-500,000, and >500,000 copies/mL) to assess the longitudinal kinetics of viral clearance. Their multivariable models demonstrated that the longest time to achieve an undetectable viral load and the lowest overall virological success rates at week 48 were concentrated within the highest stratum (>500,000 copies/mL) [11].

In our study, when virological suppression trajectories were compared across the baseline strata, a clear parallel with these findings emerged. At month six, the proportion of patients achieving an undetectable viral load was 97.6% in the low baseline viral load group, 73.3% in the high group, and 54.5% in the very high group, representing a statistically significant difference in early viral clearance kinetics (p<0.001). However, our longitudinal data demonstrate that this initial disparity progressively narrows over an extended treatment duration. By month 12 (p=0.06) and month 24 (p=0.16), the rates of virological suppression became statistically comparable among all three strata. Collectively, these data indicate that a high or very high pre-treatment viral load primarily drives a transient delay in early virological clearance at month six. Crucially, while the lack of routine post-treatment resistance testing warrants a cautious interpretation, this initial kinetic delay may not necessarily indicate early therapeutic failure or emergent resistance; rather, it underscores the clinical necessity of maintaining the regimen and closely tracking these high-viremia patients until month 12 before considering modifications.

This observation is exemplified by the single patient in our cohort who maintained a persistent but steadily declining low-level detectable viremia during the longitudinal follow-up. This individual presented with an extreme baseline viral burden of 3,393,215 copies/mL, which demonstrated gradual decay kinetics under E/C/F/TAF therapy, decreasing to 114 copies/mL at month 12 and further declining to 54 copies/mL at month 24. Despite this delayed clearance, the patient exhibited near-perfect treatment adherence, experienced no adverse events, and pre-treatment genotypic resistance testing confirmed the complete absence of baseline mutations. Because the viral load successfully and continuously decayed toward the undetectable threshold, post-treatment resistance testing was deferred. This specific outcome is strongly associated with the massive initial viral burden and slower viral clearance kinetics rather than a failure of drug potency. Notably, routine clinical follow-up extending beyond the formal study protocol at month 48 revealed that this patient eventually achieved complete virological suppression. This long-term outcome clinically confirms that the low-level viremia at month 24 represented an exceptionally prolonged viral clearance trajectory rather than true therapeutic failure.

Accumulating evidence indicates that drug potency, strict treatment adherence, resistance profiles, age, baseline viral dynamics, and baseline CD4+ T cell counts interact synergistically to determine the rate of viral suppression [11]. Multiple cohorts have established that a high baseline viral load is an independent risk factor for delayed viral suppression or true virological failure [11-14]. It has been documented that the emergence of resistance mutations after 144 weeks of E/C/F/TAF therapy is exceptionally rare [15]. In a notable study by Sax et al. exploring E/C/F/TAF efficacy specifically in naive patients with a baseline HIV-1 RNA ≥1,000,000 copies/mL, virological suppression (<50 copies/mL) was attained at week 48 in 78% (seven out of nine) of the cases. The single patient in their cohort who experienced true virological failure demonstrated poor treatment adherence during the first two months of therapy, which directly precipitated the emergence of new resistance mutations that were absent at baseline [16].

Intriguingly, our real-world results compare highly favorably with these clinical trial data; within our identical very high viral load stratum (≥1,000,000 copies/mL), 90.9% (10 out of 11) of the patients achieved complete virological suppression by month 12 (corresponding to week 48). This observation strongly corroborates the findings of Lepik et al., who demonstrated that suboptimal treatment adherence and low baseline CD4+ T cell counts are the primary drivers of emergent resistance mutations, irrespective of whether raltegravir, elvitegravir, or dolutegravir is prescribed [17]. Patients who present late with high or very high viral loads accompanied by a severely depleted immune reserve are historically at the highest risk for poor adherence, clinical complications, and virological failure [18]. In our very high viral load cohort, despite a profoundly depressed mean baseline immune status (CD4+ T cell count: 238.4±224.9 cells/µL), high adherence effectively mitigated these risks, resulting in a remarkable 90.0% suppression rate at month 24.

Our study is subject to several limitations that warrant consideration. First, the total sample size is relatively small, and the absence of a comparator control group limits our ability to definitively attribute outcomes solely to E/C/F/TAF. Second, the retrospective, single-center design introduces potential selection bias and may reflect localized clinical practices, restricting broader generalizability. Third, although our multivariable analysis adjusted for key baseline covariates such as chronological age, baseline CD4+ T cell count, and opportunistic infections, other specific baseline comorbidities and concomitant medications were not evaluated within the statistical models. Regarding the latter, while detailed registries of all concomitant medications were not systematically captured due to the retrospective design, it was clinically verified that no patients were receiving medications with known drug-drug interactions with E/C/F/TAF, ensuring that treatment efficacy and safety evaluations were not compromised. Lastly, due to the real-world observational nature of the study, post-treatment genotypic resistance testing was not routinely performed for the majority of the cohort. Nevertheless, despite these structural limitations, our study provides highly granular, longitudinal 24-month real-world evidence demonstrating the durable, long-term efficacy and safety of E/C/F/TAF in a vulnerable patient population characterized by extreme baseline viral replication.

## Conclusions

In conclusion, our study indicates that E/C/F/TAF is associated with durable long-term virological suppression and significant CD4+ T cell recovery in treatment-naive PLWH, including those presenting with high or very high baseline viral loads. Although extreme baseline viremia is associated with a transient delay in virological suppression at month six, this initial kinetic delay should not be interpreted as therapeutic failure or emergent drug resistance. Instead, these patients should be closely monitored and maintained on the regimen until at least month 12, by which time high rates of catch-up suppression are achieved. Consequently, E/C/F/TAF may serve as a viable alternative therapeutic option for individuals presenting with high baseline viral loads in clinical settings or countries where second-generation integrase inhibitor-based regimens, such as bictegravir/emtricitabine/tenofovir alafenamide or dolutegravir-based combinations, are not available or accessible. Despite the localized nature of the cohort, these longitudinal data describe a specific real-world experience regarding E/C/F/TAF clearance kinetics in high-baseline settings.
